# Delayed Diagnosis of Acute Rheumatic Fever in a Patient with Multiple Emergency Department Visits

**DOI:** 10.1155/2018/9467131

**Published:** 2018-06-04

**Authors:** Inna Kaminecki, Renuka Verma, Jacqueline Brunetto, Loyda I. Rivera

**Affiliations:** Department of Pediatrics, The Unterberg Children's Hospital at Monmouth Medical Center, Long Branch, NJ, USA

## Abstract

While the incidence of acute rheumatic fever (ARF) in the United States has declined over the past years, the disease remains one of the causes of severe cardiovascular morbidity in children. The index of suspicion for ARF in health care providers may be low due to decreasing incidence of the disease and clinical presentation that can mimic other conditions. We present the case of a 5-year-old boy with a history of intermittent fevers, fatigue, migratory joint pain, and weight loss following *group A Streptococcus* pharyngitis. The patient presented to the emergency department twice with the complaints described above. On his 3rd presentation, the workup for his symptoms revealed the diagnosis of acute rheumatic fever with severe mitral and aortic valve regurgitation. The patient was treated with penicillin G benzathine and was started on glucocorticoids for severe carditis. The patient was discharged with recommendations to continue secondary prophylaxis with penicillin G benzathine every 4 weeks for the next 10 years. This case illustrates importance of primary prevention of acute rheumatic fever with adequate antibiotic treatment of *group A Streptococcus* pharyngitis. Parents should also receive information and education that a child with a previous attack of ARF has higher risk for a recurrent attack of rheumatic fever. This can lead to development of severe rheumatic heart disease. Prevention of recurrent ARF requires continuous antimicrobial prophylaxis. Follow-up with a cardiologist every 1-2 years is essential to assess the heart for valve damage.

## 1. Introduction

Acute rheumatic fever is an immune-mediated consequence of *group A Streptococcus* pharyngitis.

The incidence of ARF in the United States and Western Europe has decreased markedly during the last 50 years [1]. The current annual incidence of ARF in the continental United States is approximately 0.04–0.06 cases per 1,000 children with most cases in children with 5 to 15 years of age [2]. Children with ARF present with one or more of the following features: fever, carditis, polyarthritis, chorea, erythema marginatum, and subcutaneous nodules. Laboratory findings include elevated inflammatory markers and elevated/rising antistreptolysin O titer or antideoxyribonuclease B antibodies [3]. Symptoms of ARF develop in two to four weeks following untreated or inadequately treated pharyngitis. Recurrent or severe acute rheumatic fever can be associated with permanent damage of the cardiac valves and development of rheumatic heart disease, leading to severe cardiovascular morbidity and mortality in children [4].

## 2. Case Presentation

A 5-year-old boy was hospitalized with a 3-month history of intermittent fevers, fatigue, migratory joint pain, and weight loss. Three months ago, the patient was diagnosed with *group A Streptococcus* pharyngitis and was prescribed a 10-day course of amoxicillin. Two weeks later, he started having pain in his knees, elbows, shoulders, and neck. The patient was diagnosed with influenza and completed a course of oseltamivir. His joint pain and intermittent fever persisted. He began to develop malaise, weakness, and difficulty waking and was noted to have cough. During the next month, the patient was evaluated twice in different emergency departments with the same complaints. Evaluation at the emergency department revealed a mildly elevated C-reactive protein and erythrocyte sedimentation rate. Chest X-ray findings were consistent with pneumonia, and he was prescribed amoxicillin/clavulanic acid. Blood and urine cultures were negative. Three weeks later, the patient presented to his pediatrician with complaints of bilateral ear pain. His fevers, joint pain, difficulty walking, and fatigue persisted. During physical examination, the pediatrician noted a new systolic murmur. It prompted his hospitalization for further evaluation and management.

On examination, the patient appeared unwell, but nontoxic. His temperature was 96.8°F (36.0°C), heart rate was 123 beats/min, respiratory rate was 23/min, blood pressure was 109/65 mm Hg, and oxygen saturation was 98% on room air. His growth was plotted on the 25th percentile for weight and height. He had a V/VI holosystolic murmur with thrill at the mitral area with radiation to the axilla and interscapular area. The boy reported pain during active and passive motion of both knees and the neck. His left knee was slightly swollen. Examination of the skin revealed presence of small (1 centimeter in diameter) painless, firm nodules over extensor surfaces of ankles and elbows bilaterally, consistent with subcutaneous nodules.

**Figure 1:**
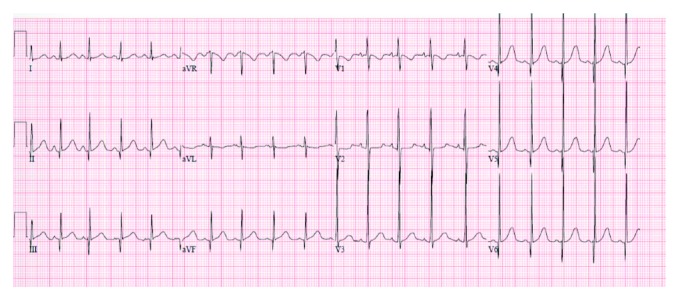
12-lead electrocardiogram with increased R wave voltage above 98th percentile for age in leads V5 and V6 and Q wave in lead V6 above 98th percentile for age.

**Figure 2:**
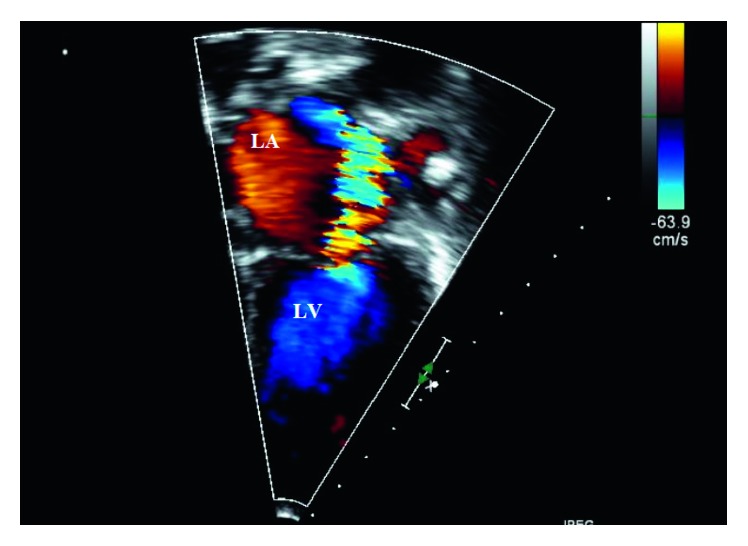
Transthoracic echocardiogram in apical four-chamber view demonstrating enlargement of the left atrium (LA) and severe mitral valve regurgitation.

Further review of the patient's history of present illness reveled noncompliance with previously prescribed treatment. Parents reported that the boy refused to take antibiotics. Laboratory evaluation showed the white blood cell count of 9.9 × 10^3^ *μ*/L (9.9 × 10^9^/L), platelet count of 495 × 10^3^/*µ*L (495 × 10^9^/L), hemoglobin level of 11.2 g/dL (112 g/L), C-reactive protein level of 84.3 mg/L (802 nmol/L), and erythrocyte sedimentation rate of 93 mm/hr. Antistreptolysin O titers were 4,133 IU/ml (reference range, <150 IU/ml). Results of urinalysis were normal. Electrocardiography showed normal sinus rhythm with signs of left ventricular hypertrophy with volume overload with increased R wave voltage above 98th percentile for age in leads V5 and V6 and Q wave in lead V6 above 98th percentile for age (Figure 1). Echocardiographic findings included moderate left atrial enlargement, severe mitral valve regurgitation (Figure 2), and aortic valve regurgitation. The PR interval was within a normal limit of 0.120 seconds for 5-6-year-old children.

Based on the history of preceding *group A Streptococcus* infection, clinical symptoms, elevated acute phase reactants, and echocardiographic findings, the patient was diagnosed with acute rheumatic fever. This patient received intramuscular penicillin G benzathine and was started on glucocorticoids for severe carditis. He also was prescribed aspirin for symptomatic management of arthritis with an initial dose at 75 mg/kg per day in divided doses every 6 hours during two days of hospitalization. The dose of aspirin at discharge was decreased to 50 mg/kg per day in divided doses every 8 hours. The patient was discharged with recommendations to continue secondary prophylaxis with penicillin G benzathine every 4 weeks for the next 10 years.

## 3. Discussion

The diagnosis of acute rheumatic fever historically was always made using Jones criteria [3]. Recently, the Jones criteria were modified in the year 2015 by the World Heart Association, with emphasis on the role of Doppler echocardiography in the diagnosis of ARF [5]. The diagnosis of initial ARF for low-risk populations is based on the presence of 2 major criteria or 1 major and 2 minor criteria.

  Major manifestations: carditis (clinical and/or subclinical), arthritis, chorea, subcutaneous nodules, and erythema marginatum.
  Minor manifestations: polyarthralgia, fever ≥ 38.5C, ESR ≥ 60 mm in the first hour and/or CRP ≥ 3 mg/dL, and prolonged PR interval.

Confirmation of previous *group A Streptococcus* pharyngitis is required for diagnosis and includes elevated or rising antistreptolysin O titer or antideoxyribonuclease B antibodies, positive throat culture for *group A Streptococci*, and positive rapid *group A Streptococcal* test in a child with clinical presentation of streptococcal pharyngitis [6].

This patient presented to our facility with 3 major (carditis, arthritis, and subcutaneous nodules) and 1 minor (ESR ≥ 60 and/or CRP ≥ 3 mg/dL) criteria which lead us to the diagnosis of ARF. Although the patient was evaluated twice in different emergency departments, carditis was not identified on physical examination. The presence of a new murmur on auscultation by the primary care provider raised suspicion for ARF and prompted further evaluation. Classically, as discussed in the 1992 revised Jones criteria statement, carditis as a major manifestation of ARF has been a clinical diagnosis based on the auscultation on murmurs that indicate mitral or aortic valve regurgitation [3]. New recommendations emphasize the use of echocardiography/Doppler studies in the diagnosis of subclinical carditis, as auscultatory findings might be absent or not recognized by the provider [7, 8]. Doppler findings of subclinical carditis are presented in Table 1.

Another common presentation of ARF is migratory polyarthralgia with involvement of large joints, such as knees, elbows, and ankles. About 35–66% of children with ARF have symptoms of polyarthritis [7, 9]. Polyarthralgia is not exclusive to ARF and can be observed in juvenile idiopathic arthritis, reactive arthritis, systemic lupus erythematosus, and mixed connective tissue disease. In the case of our patient, it is possible that the history was limited due to the patient's age and the pain in his joints was interpreted as muscle pain due to influenza infection.

Skin findings in ARF include a unique serpiginous rash over the trunk or extremities (erythema marginatum) and subcutaneous painless nodules (SCN). SCN are usually found on the extensor surfaces of knees, elbows, wrists, the occipital area, and along the spinous processes of the thoracic and lumbar vertebrae. Subcutaneous nodules are rarely observed in patients with ARF, and their presence is usually associated with carditis [10]. This emphasizes the importance of detailed physical examination in children with suspected ARF.

Involvement of the central nervous system in patients with ARF manifests as Sydenham chorea. Chorea is characterized by abrupt and involuntary movements of the trunk and/or extremities. It is also very often associated with emotional lability and features of obsessive-compulsive disorder [11]. Chorea often occurs many months after the symptoms of *group A Streptococcus* pharyngitis and may present as an isolated finding. That is why the presence of Sydenham chorea alone is adequate to make a diagnosis of ARF.

Management of ARF includes intramuscular administration of long-acting penicillin G benzathine for eradication of *group A Streptococci* [6, 12]. Symptomatic management of arthritis includes administration of nonsteroidal anti-inflammatory medications. Low-dose glucocorticoids can be used for patients who are allergic to aspirin and in patients with severe carditis [13]. Chorea in ARF is usually self-limited. Administration of carbamazepine or valproic acid can be considered in patients whose symptoms interfere with activities of daily living [14]. All patients who have had acute rheumatic fever will require secondary prophylaxis with intramuscular penicillin G benzathine every 4 weeks for 5–10 years [6, 15]. Duration of prophylaxis depends on severity of the disease. Patients with valve lesions and signs of heart failure might require valve surgery.

## 4. Conclusion

Acute rheumatic fever has a low incidence in the United States but should be considered in patients with evidence of previous *group A Streptococcus* pharyngitis and clinical symptoms of acute rheumatic fever such as carditis, arthritis, chorea, subcutaneous nodules, and erythema marginatum. Echocardiography with Doppler should be performed in all cases of confirmed or suspected acute rheumatic fever. It is essential that parents and patients receive adequate information about ARF and rheumatic heart disease. Caregivers should be educated that treatment of *group A Streptococcus* pharyngitis is crucial for preventing acute rheumatic fever. This requires prompt diagnosis and treatment with a full course of antibiotics. If prophylaxis was initiated late or was discontinued too early, it can lead to acute rheumatic fever with risk of development of persistent valve damage. After a first episode of rheumatic fever, it is essential to prevent development of recurrent attack of ARF, as it can cause further cardiac valve damage. Risk of recurrence of rheumatic fever also depends on multiple factors. The factors that predispose to acquiring *group A Streptococcus* pharyngitis include children living in crowded situations (overcrowding in the house and college dormitories). Secondary prophylaxis includes administration of continuous antimicrobial prophylaxis to patients with ARF in order to prevent further damage of the valve that can lead to severe rheumatic heart disease. The duration of prophylaxis depends on whether residual valve damage is present or absent [6]. The follow-up with a cardiologist is crucial for monitoring patients with ARF and rheumatic heart disease.

## Figures and Tables

**Table 1:** Doppler and morphological findings in rheumatic valvulitis.

Doppler findings	Morphological findings
(i) Pathological mitral regurgitation (all 4 criteria should be met)	(i) Acute mitral valve changes
(1) Seen in at least 2 views	(1) Annular dilation
(2) Jet length ≥ 2 cm in at least 1 view	(2) Chordal elongation
(3) Peak velocity > 3 m/s	(3) Anterior/posterior leaflet tip prolapse
(4) Pansystolic jet in at least 1 envelope	(4) Chordal rupture
(ii) Pathological aortic regurgitation (all 4 criteria should be met)	(5) Beading/nodularity of leaflet tips
(1) Seen in at least 2 views	(ii) Chronic mitral valve changes
(2) Jet length ≥ 1 cm in at least 1 view	(1) Leaflet thickening
(3) Peak velocity > 3 m/s	(2) Chordal thickening and fusion
(4) Pandiastolic jet in at least 1 envelope	(3) Restricted leaflet motion
	(4) Calcification
	(iii) Aortic valve changes in acute or chronic carditis
	(1) Irregular or focal leaflet thickening
	(2) Restricted leaflet motion
	(3) Leaflet prolapse
	(4) Coaptation defect

*Note*. Reprinted from “Revision of the Jones criteria for the diagnosis of acute rheumatic fever in the era of Doppler echocardiography: a scientific statement from the American Heart Association” by Gewitz et al. [5].
